# Pathway importance by graph convolutional network and Shapley additive explanations in gene expression phenotype of diffuse large B-cell lymphoma

**DOI:** 10.1371/journal.pone.0269570

**Published:** 2022-06-24

**Authors:** Jin Hayakawa, Tomohisa Seki, Yoshimasa Kawazoe, Kazuhiko Ohe

**Affiliations:** 1 Department of Biomedical Informatics, Graduate School of Medicine, The University of Tokyo, Tokyo, Japan; 2 Department of Healthcare Information Management, The University of Tokyo Hospital, Tokyo, Japan; 3 Artificial Intelligence in Healthcare, Graduate School of Medicine, The University of Tokyo, Tokyo, Japan; Chinese Academy of Sciences, CHINA

## Abstract

Deep learning techniques have recently been applied to analyze associations between gene expression data and disease phenotypes. However, there are concerns regarding the black box problem: it is difficult to interpret why the prediction results are obtained using deep learning models from model parameters. New methods have been proposed for interpreting deep learning model predictions but have not been applied to genetics. In this study, we demonstrated that applying SHapley Additive exPlanations (SHAP) to a deep learning model using graph convolutions of genetic pathways can provide pathway-level feature importance for classification prediction of diffuse large B-cell lymphoma (DLBCL) gene expression subtypes. Using Kyoto Encyclopedia of Genes and Genomes pathways, a graph convolutional network (GCN) model was implemented to construct graphs with nodes and edges. DLBCL datasets, including microarray gene expression data and clinical information on subtypes (germinal center B-cell-like type and activated B-cell-like type), were retrieved from the Gene Expression Omnibus to evaluate the model. The GCN model showed an accuracy of 0.914, precision of 0.948, recall of 0.868, and F1 score of 0.906 in analysis of the classification performance for the test datasets. The pathways with high feature importance by SHAP included highly enriched pathways in the gene set enrichment analysis. Moreover, a logistic regression model with explanatory variables of genes in pathways with high feature importance showed good performance in predicting DLBCL subtypes. In conclusion, our GCN model for classifying DLBCL subtypes is useful for interpreting important regulatory pathways that contribute to the prediction.

## Introduction

Gene expression patterns are associated with cell function, and vary among tissues. Specific genes are expressed in various diseases, some of which are prognostic or therapeutic targets. Various statistical approaches including machine learning have been attempted to reveal the associations between gene expressions and disease phenotypes. Deep learning is an emerging machine learning method that enables highly accurate predictions of these associations. However, it is difficult to interpret these predictions from the model parameters. The problem is often referred to as the black box problem in deep learning [1]. Explainability is crucial for predictive models in medicine and other research fields. Several methods have been used to explain such models, such as the local interpretable model-agnostic explanations [2], DeepLIFT [3, 4], layer-wise relevance propagation [5], and classic Shapley value estimation [6–8]. SHapley Additive exPlanations (SHAP) [9] is an improved method compared to classic Shapley value estimation, because it ensures local accuracy (accurate explanation of a model prediction for each input) and consistency (magnitude of the correlation of feature contributions among models is consistent); however, its utility for evaluating gene expression levels has not yet been evaluated in detail.

In genetics, Gene Set Enrichment Analysis (GSEA) [10, 11] has been applied to gain insights into the functional associations of phenotypes and gene sets. Genes in GSEA are grouped into gene sets and analyzed together, which enhances the sensitivity of the analysis compared to that of single gene analysis. Gene sets of genetic pathways, cytogenetic bands, and gene ontology have been manually curated from the databases. Genetic features such as expression, mutation, and copy numbers were compared between two phenotypes to obtain enrichment scores, which were calculated from a correlation of genetic features and phenotypes and Kolmogorov–Smirnov statistics. Next, p-values and the false discovery rate (FDR) were obtained via permutation. GSEA has revealed highly expressed gene sets associated with phenotypes when gene expression was used as a feature. However, high expression is not the only characteristic of the correlation between gene expression patterns and phenotypes. A critic of GSEA argued that the correlation structure was not considered because it detected gene expression in a gene set that was biased toward a specific class [12]. Some complex relationships between features and phenotypes are ignored by GSEA, whereas deep learning can use these relationships when making predictions. By interpreting the predictions of deep learning, it may be possible to detect associations between gene expression patterns and phenotypes that are not detected using GSEA. We used SHAP to interpret the deep learning model predictions because this method can summarize the feature importance across datasets. SHAP estimates the feature contribution, or Shapley value, to prediction on an additive scale. A Shapley value on a feature which greatly contributes to predicting a label takes a high value even if the correlation between the feature and the label is negative or complex. Although SHAP has been validated in many machine learning models, it has not been established for genetic pathway analysis. Therefore, we investigated a method to estimate the Shapley values of the genetic pathways in this study. We hypothesized that Shapley values from an intermediate layer of a deep learning model that contains genetic pathway networks corresponds to the feature importance of genetic pathways in phenotype prediction. A graph convolutional network (GCN) was used to implement this network in this study. GCN is a deep learning method that utilizes the relationship between variables, such as social networks [13] and protein-protein interaction networks [14]. Recently, some studies reported the high performance of GCN analysis using a graph of protein-protein interaction networks for phenotype prediction of cancer types [15] and breast cancer subtypes [16] from gene expression profiles.

In this study, we examined the effects of SHAP on genetic pathways using a GCN classification model to explore the possibility of selecting pathways that contribute to classifying cancer subtypes. We retrieved diffuse large B-cell lymphoma (DLBCL) datasets containing microarray gene expression data and gene expression subtypes [17] from the Gene Expression Omnibus (GEO) database [18]. DLBCL has two subtypes, the germinal center B-cell-like (GCB) type and activated B-cell-like (ABC) type. These subtypes are derived from different maturation stages of B cells and exhibit different gene expression patterns. The prognosis of patients with DLBCL treated with standard combination chemotherapy differs by subtype, and an optimal treatment strategy is being developed [19, 20]. Recent studies revealed the molecular characteristics and detailed gene clusters associated with these subtypes which differ in prognosis [21–23]. Understanding the genetic function of DLBCL is important for investigating future treatment options. First, we constructed a GCN model that classified DLBCL subtypes based on microarray gene expression profiles [24]. Next, the feature importance of the gene sets corresponding to genetic pathways on the prediction was obtained using SHAP. The feature importance was compared to the results of GSEA.

## Methods

### Dataset

Gene expression data and clinical information in GSE31312 [25] and GSE10846 [19] were obtained from the GEO database [18]. Each dataset included 498 and 414 patients with DLBCL, respectively. The same microarray platform (Affymetrix Human Genome U133 Plus 2.0 Array, Santa Clara, CA, USA) was used for all gene expression data. In each study, the patients with DLBCL subtypes [24] were labeled as GCB, ABC, or unclassifiable in gene expression profiling. The probabilities for each class were estimated using the Bayesian classifier [19, 26, 27]. The samples were classified as unclassifiable when the predicted probability did not exceed the threshold.

Microarray RAW data were downloaded from the database. Each data set was normalized using robust multichip analysis [28] to obtain true signal intensities and to eliminate noise for each probe. R 3.6.2 was used for normalization (The R Project for Statistical Computing, Vienna, Austria). Next, the probe-level signal intensities were assigned to the corresponding genes. The values were log2 transformed with 1 as the cutoff, resulting in a close to normal distribution. The gene expression levels in each sample were standardized with a mean of 0 and a variance of 1. The expression levels of 4816 genes across 186 pathways were included in Kyoto Encyclopedia of Genes and Genomes (KEGG) pathways [29] (c2.cp.kegg.v7.3.symbols.gmt) in MSigDB [10, 11] registered at the Broad Institute and were selected as explanatory variables for our classification models. Genes in the KEGG pathways, but not in the microarray data set, were assigned a value of 0. The number of labels should be reduced to 2 to compare the results of SHAP and GSEA. Therefore, the samples labeled as GCB or ABC were used as objective variables, and those labeled as unclassifiable were excluded from analysis. There were 227 GCB samples and 199 ABC samples among the 426 cases in GSE31312, and 183 GCB samples and 167 ABC samples among the 350 cases in GSE10846. The datasets were assigned as the training and test datasets according to the available sample size. The amount of data on gene expression levels used for input to the model was large (4816 gene expression levels), making it difficult to converge the learnable parameters when the sample size of the training dataset was small. Therefore, GSE31312 was assigned to the training dataset to train the models, and GSE10846 was assigned to the test dataset to evaluate the performance of the models.

### Proposed model

A classification model was created to classify samples into two classes, GCB and ABC, based on the gene expression levels obtained during preprocessing. A GCN was constructed for this model. To create graphs representing genetic pathways, we first selected 186 pathways annotated in the KEGG [29] pathways (c2.cp.kegg.v7.3.symbols.gmt) in MSigDB [10, 11] and the genes included in these pathways. Therefore, important pathways determined using the GCN and enriched pathways identified using GSEA are equivalent and can be compared. We set the nodes corresponding to the genes. A mean of 69 genes was identified in the 186 KEGG pathways (S1A Fig). Next, the relationships between the genes in the KEGG pathways were determined from the KEGG website and were set as edges between the nodes. Using these nodes and edges, we constructed 186 graphs G=(V,E), each representing a KEGG pathway. The graphs contained 12,797 nodes and 146,343 edges, including duplications among the different pathways. Graph convolutions were conducted assuming undirected graphs. The edge density, which is the percentage of edge numbers among the possible edge combinations in a graph, showed an average value of 0.15 and standard deviation (SD) of 0.13 (S1D Fig). We used an adjacency matrix $A_{k}\in R^{N_{k}\times N_{k}}$ of the graphs to perform graph convolution [13] for the gene expression of each graph, where *A*_*k*_ was the adjacency matrix of the *k*th graph and *N*_*k*_ was the number of nodes in the *k*th graph. We did not assume a continuous edge weight, and thus all elements in the adjacency matrix were either 0 (no edge) or 1 (with an edge). In the GCN, the following propagation was performed for each layer:

$$H^{\left(I+1\right)}=\sigma ({\tilde{D}}^{-\frac{1}{2}}\tilde{A_{k}}{\tilde{D}}^{-\frac{1}{2}}H^{\left(l\right)}W^{\left(l\right)})$$

$\tilde{A_{k}}$ is the matrix sum of the adjacency matrix and identity matrix, where $\tilde{A_{k}}=A_{k}+I_{N_{k}}.\;I_{N_{k}}$ is an *N*_*k*_×*N*_*k*_ identity matrix. $\tilde{D}$ is a matrix for normalization with ${\tilde{D}}_{ii}=\sum_{j}{\tilde{A}}_{ij}.\;W^{\left(l\right)}$ is the updatable weight matrix. H^(*l*)^ is the output of the *l*th layer (H^(0)^ = *X*), where *X* is the matrix for gene expression. *σ*(∙) is the activation function, and rectified linear unit (ReLU) was adopted. ReLU is an activation function defined to output the following value for argument *x*.


$$f(x)=\left\{ \begin{array}{l} 0\;(x\leq 0)\\ x\;(x>0) \end{array}\right.$$

**Fig 1:**
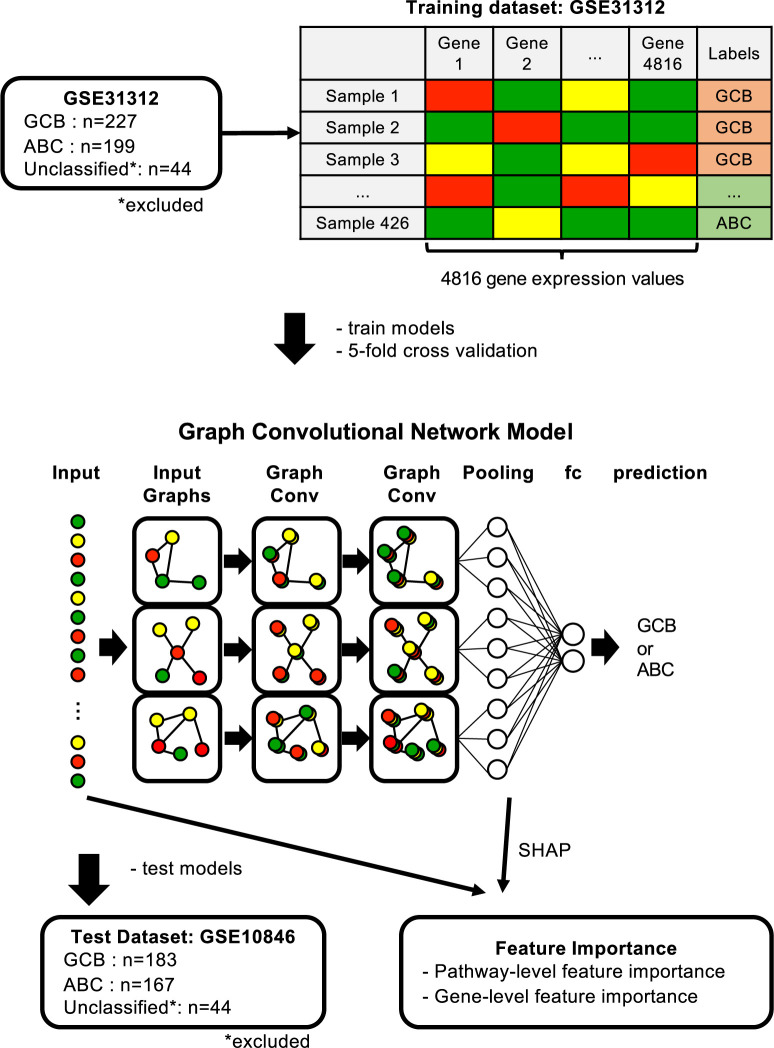
Scheme of data preparation, training and testing the models, and obtaining feature importance. Illustration of the scheme in this study. GSE31312 and GSE10846 were used as training and test datasets and preprocessed and shaped into *S* (*samples*)×*N* (*genes*) tables. The input layer of the graph convolutional network (GCN) model had N gene expression levels for each sample. Expression levels were input to corresponding nodes in the graph of genetic pathways with nodes of the genes and edges of the genetic interactions based on KEGG pathways. Nodes were processed twice by graph convolution, and then passed through an average pooling layer. The fully connected layer was used to classify the two phenotypes. The model was trained using the training dataset, and then evaluated using the test dataset. The feature importance in the GCN model was obtained using Shapley Additive exPlanations (SHAP).

There were two graph convolution layers in the GCN model, followed by an average pooling layer. A node was updated from its own features and from the features of its neighbors through the graph convolution. The GCN had two graph convolution layers, demonstrating that a node was updated by the local features of the nodes traced by two edges. A node in the pooling layer received outputs from the nodes in the corresponding graph. Next, 10 output values per graph, in a total of 1860 dimensions, were obtained as the output of the average pooling layer. The output of the average pooling layer was linearly transformed in the next fully connected layer, and the outputs were transformed in the softmax layer to obtain the probability of classification into two classes, GCB and ABC (Fig 1). We used a multilayer perceptron (MLP) model with three fully connected layers and a softmax layer, as well as a GCN-MLP model with three fully connected layers between a GCN pooling layer and softmax layer for comparison with the GCN model. Dropout and batch normalization were performed following each graph convolution and linear transformation. The loss function was cross-entropy loss with L2 regularization, and was weighted according to the number of samples in each class. An Adam [30] optimizer was used to train the models. Adam is a gradient-based optimizer of stochastic objective functions that efficiently works with non-convex optimization by computing adaptive learning rates for parameters from estimates of the first and second moments of the gradients. Early stopping terminated the model training when the loss function did not decrease five times.

A random search was performed, and hyperparameters were determined by five-fold cross-validation. The classification performance on the training dataset was obtained from the average classification performance on the validation set in five-fold cross-validation. The best model with the highest accuracy was selected to evaluate evaluation the test dataset. The cutoff value of the model output was determined as the point at which Youden’s index (sensitivity + specificity -1) was maximized from the receiver operating characteristics curve on the training dataset. We determined the classification performance for the test dataset using the trained model and cutoff value. As a measure of classification performance, accuracy was the percentage of correct predictions among all predictions, precision was the probability that the predicted label was correct, recall was the probability that the prediction was correct for the correct label, and F1 score was the harmonic mean of precision and recall. Micro-averages [31] were used for each measure.

### Feature importance

The importance of the variables that contributed to prediction of the two classes was studied using the trained model and training dataset. We computed Shapley values for each variable to estimate the feature importance. Shapley values were additive scale measures that represented the attribution of variables to the prediction. Shapley values were computed using the SHAP package in Python [9]. Positive and negative Shapley values indicate that a variable positively and negatively contributes to the prediction of a class, respectively. Because there were only two objective variables, GCB or ABC, the Shapley values for the model indicated the degree of the contribution to distinguish between GCB and ABC. The magnitude of Shapley values is consistent across samples and variables, such that the feature importance can be summarized by calculating the mean absolute Shapley values across samples [9]. The importance of pathways was determined from the Shapley values of the output of the pooling layer after graph convolution. Because there were multiple outputs from each pathway after the pooling layer and a class to which each output contributed may differ, simple summation of the pathway outputs did not indicate the class in which a pathway contributed to a prediction. Therefore, we examined the absolute Shapley values for the prediction. We obtained Shapley values from the pooling layer, and absolute Shapley values were averaged for each graph. These values indicate the feature importance of the pathways. They were compared between pathways, and ranked according to their feature importance.

The correlation of DLBCL subtypes and gene sets in the training dataset was estimated using GSEA software version 4.1.0 [10, 11]. KEGG pathways (c2.cp.kegg.v7.3.symbols.gmt) were adopted for gene sets so that the same pathways containing the same genes were used in SHAP and GSEA. Normalized enrichment scores for each KEGG pathway were computed from Kolmogorov-Smirnov statistics and used as statistics to compare the degree in which phenotypes a gene set was overexpressed across gene sets. The P-values and FDR were obtained for each pathway, where FDR < 0.25 was set as the cutoff.

Therefore, the pathways with high importance in the SHAP and pathways selected by GSEA were compared. As SHAP did not produce statistics on significance, the mean absolute Shapley values of the pathways were sorted in descending order, and the same number of pathways was selected from the SHAP and GSEA results. In addition, Shapley values for each expression of each gene were obtained and ranked according to the mean absolute Shapley values.

To confirm that the outputs from the pathways with high mean absolute Shapley values had high potential to classify the phenotypes of gene expression profiling, we used logistic regression classifiers, by repeating the phenotype predictions with selected genes. First, we selected the genes included in all five pathways in descending order of their Shapley values. Second, using a logistic regression model with the genes as explanatory variables, phenotype predictions of the DLBCL subtype were performed. L2 regularization was used to avoid diverting the parameters because of the strong correlation of genes in the same pathways. The training dataset was used for model training, and the classification performance was measured using the test dataset. We also compared the classification performance with every 100 genes in descending order of the Shapley value and genes in five pathways with the highest absolute normalized enrichment scores.

The Pytorch 1.1, Deep Graph Library [32], and SHAP [9] as Python 3.8 packages were used for implementation. All analyses were performed on NVIDIA Tesla V100 GPU with 16GB memory. This study was approved by the institutional ethics committee (Approval Number: 2019263NI) and conducted in accordance with the Declaration of Helsinki.

## Results

First, the classification performance of the GCN model was compared with that of the MLP model and GCN-MLP models. The network in the GCN model was constructed to represent gene associations in the KEGG pathways [29]. We trained the models using the training dataset from GSE31312 [25] and evaluated the performance using the test dataset from GSE10846 [19]. The numbers of trainable parameters for each model were 3,865 for the GCN, 4,735,680 for the GCN-MLP, and 6,827,000 for the MLP. The GCN model showed an accuracy of 0.965, precision of 0.960, recall of 0.965, and F1 score of 0.962 for the average classification performance in the five-fold cross-validation, and an accuracy of 0.914, precision of 0.948, recall of 0.868, and F1 score of 0.906 for the test dataset. The accuracies of the other models on the test dataset were 0.857 and 0.869 for the MLP and GCN-MLP models, respectively. The prediction performance of each model is presented in Table 1. The GCN showed the best accuracy among the tested deep learning methods.

**Table 1:** Parameters and classification performance of each model.

		Training dataset ^a^				Test dataset ^b^			
	Parameters	Accuracy	Precision	Recall	F1 score	Accuracy	Precision	Recall	F1 score
**MLP**	6,827,000	0.979	0.970	0.985	0.978	0.857	0.770	1.000	0.870
**GCN**	3,865	0.965	0.960	0.965	0.962	0.914	0.948	0.868	0.906
**GCN-MLP**	4,735,680	0.965	0.947	0.980	0.963	0.869	0.817	0.934	0.872

^a^ Classification performance on the training dataset was the average performance on validation sets in five-fold cross-validation.

^b^ Model training was stopped at 17 epochs in MLP, 16 epochs in GCN, and 16 epochs in GCN-MLP.

MLP: multilayer perceptron, GCN: graph convolutional neural network

**Fig 2:**
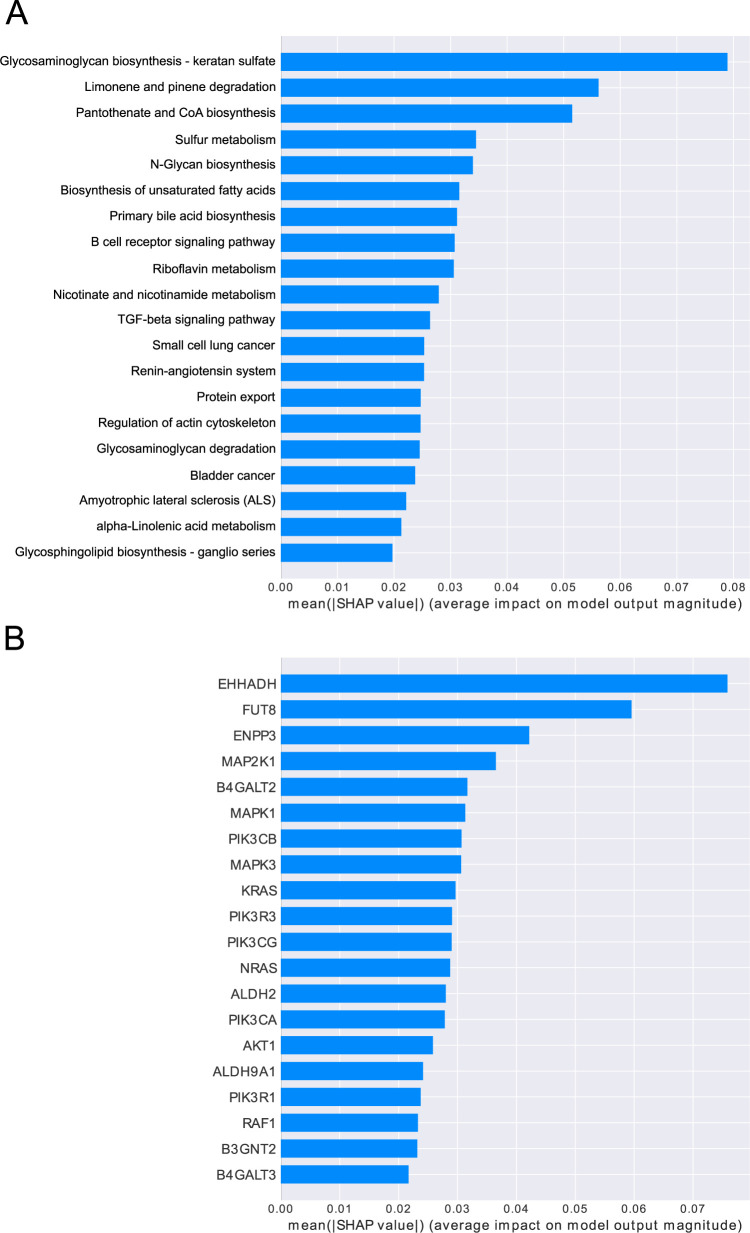
SHAP results of intermediate and input layers of the graph convolution network. A: Feature importance of the pathways for prediction, sorted in descending order. Each bar shows the mean absolute Shapley values of each pathway in the output of the pooling layer. B: Feature importance of the gene expression levels on the prediction, sorted in descending order. Each bar shows the mean absolute Shapley values for the gene expression levels.

**Fig 3:**
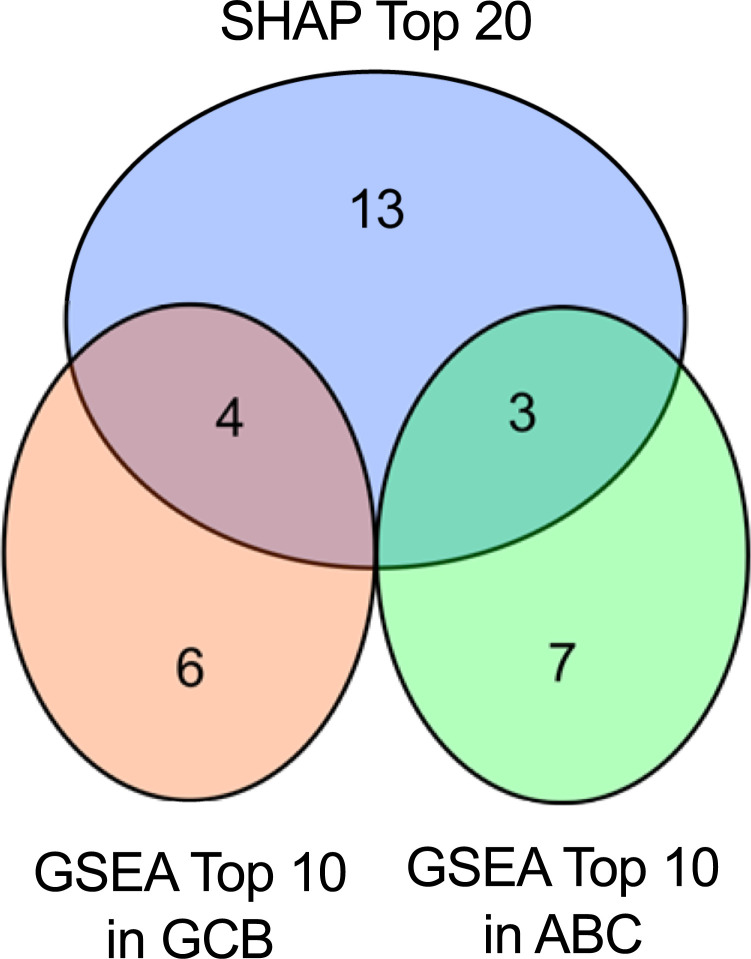
Overlap of important pathways in SHAP and GSEA. Circles correspond to pathways with high feature importance in the Shapley additive explanation and were highly enriched in gene set enrichment analysis. The overlap in the two diagrams indicates the pathways commonly listed in the two methods.

To determine the feature importance of the pathways and gene expression from the trained model, SHAP was applied to the output of the pooling layer after the graph convolution layers and to the input layer. SHAP values were computed using the trained models for all pathways and all genes in 33.2 s and 3 min 39s, respectively. The absolute Shapley values were averaged for each pathway to obtain the feature importance. The feature importance values of the pathway outputs sorted in descending order are shown in Fig 2A. GSEA was performed to obtain normalized enrichment scores for the pathways by comparing GCB and ABC. The top enriched pathways in GCB and ABC are shown in Table 2. The top three KEGG pathways for each subtype according to the normalized enrichment scores in GSEA were the TGF-β signaling pathway (hsa04350), regulation of actin cytoskeleton (hsa04810), and pantothenate and CoA biosynthesis (hsa00770) in GCB; and protein export (hsa03063), N-glycan biosynthesis (hsa00510), and glycosaminoglycan biosynthesis keratan sulfate (hsa00532) in ABC. The top 20 pathways with the high mean absolute Shapley values included these six enriched pathways. The number of overlapping pathways in SHAP and GSEA are shown in Fig 3. The mean number of nodes in the 20 pathways selected by GSEA were 41 genes in SHAP and 82 genes in GSEA (S1B, S1C Fig). The mean edge densities of the 20 pathways selected by SHAP and GSEA were 0.15 (SD 0.13) and 0.11 (SD 0.11), respectively (S1E, S1F Fig). In addition, the B cell receptor signaling pathway (hsa04662), which was characteristically expressed in ABC DLBCL, showed the 8^th^ highest mean absolute Shapley values, but it was the 31^st^ enriched pathway of ABC in GSEA.

**Table 2:** Top pathways for each DLBCL subtype in gene set enrichment analysis.

**GCB**				
	**Name**	**NES**	**p-value**	**FDR q-value**
**1**	TGF beta signaling pathway	1.874	0.000	0.13
**2**	Regulation of actin cytoskeleton	1.819	0.000	0.134
**3**	Pantothenate and CoA biosynthesis	1.761	0.006	0.161
**4**	ECM receptor interaction	1.726	0.025	0.171
**5**	Dilated cardiomyopathy	1.713	0.014	0.152
**6**	Nicotinate and nicotinamide metabolism	1.712	0.006	0.128
**7**	Melanoma	1.706	0.000	0.117
**8**	Leukocyte transendothelial migration	1.673	0.000	0.133
**9**	Cell adhesion molecules CAMs	1.673	0.008	0.119
**10**	Focal adhesion	1.659	0.008	0.120
ABC				
	**Name**	**NES**	**p-value**	**FDR q-value**
1	Protein export	-1.863	0.000	0.126
2	N-Glycan biosynthesis	-1.787	0.000	0.150
3	Glycosaminoglycan biosynthesis keratan sulfate	-1.780	0.000	0.111
4	Aminoacyl tRNA biosynthesis	-1.779	0.002	0.084
5	Fatty acid metabolism	-1.752	0.006	0.091
6	Proteasome	-1.717	0.012	0.109
7	Systemic lupus erythematosus	-1.679	0.016	0.130
8	Pyrimidine metabolism	-1.673	0.006	0.119
9	RNA polymerase	-1.631	0.030	0.150
10	Spliceosome	-1.560	0.065	0.237

GCB: germinal center B-cell-like type, ABC: activated B-cell-like type, NES: normalized enrichment score, FDR: false discovery rate.

**Fig 4:**
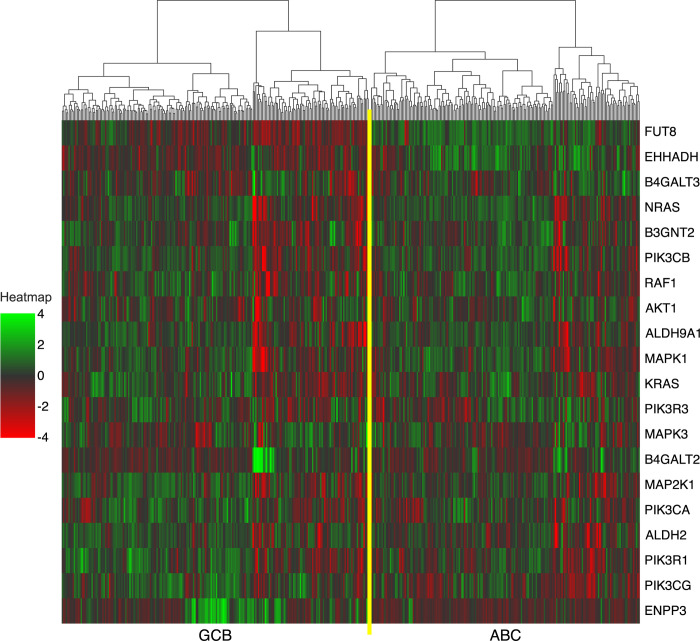
Heatmap of the top 20 genes in SHAP. Each raw read represents a single gene and each column represents a tumor sample. The top 20 genes in SHAP are ordered by the correlation coefficient with the subtypes. Samples are clustered by the gene expression levels for each subtype. The raw data of microarrays were normalized using robust multichip analysis and standardized, which are shown in the heatmap. The gradual color change from green to red represents high to low expression. Samples are ordered by subtypes; samples on the left and right of the yellow center line are the germinal center B-cell-like and activated B-cell-like types, respectively.

Regarding gene expression, *EHHADH*, *FUT8*, *ENPP3*, *MAP2K1*, and *B4GALT2* showed high Shapley values (Fig 2B). Most top-ranked genes were included in the highly important metabolic and signaling pathways. Genes related to metabolism, such as *EHHADH*, *FUT8*, and *ENPP3*, were also included in the top-ranked pathways in GSEA. However, genes involved in signal transduction, such as *MAP2K1*, were not ranked high in GSEA. A heatmap of the top 20 genes selected by SHAP is shown in Fig 4.

**Fig 5:**
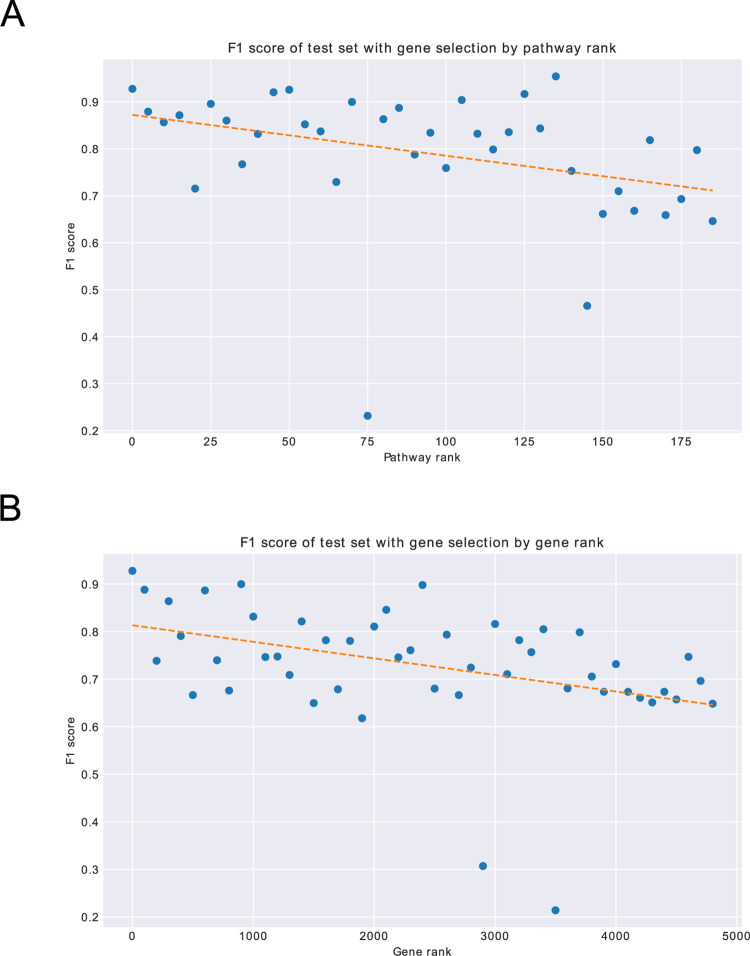
Classification performance by logistic regression with genes selected by the rank of feature importance. Classification performance using logistic regression classifiers is shown. The F1 scores for the test dataset are plotted for each model with explanatory variables of selected genes by the rank of feature importance. The dashed lines represent the linear regression line. A: Gene expression levels in the five pathways selected by the rank of feature importance of the pathway were used as explanatory variables for each logistic regression classifier. B: Gene expression levels by every 100 successive ranks of feature importance of the input were used as explanatory variables for each logistic regression classifier.

Next, the correlation of the feature importance by SHAP with classification performance was investigated using logistic regression classifiers according to the feature importance on the pathways. The DLBCL subtypes were predicted using a logistic regression classifier with the gene expression levels in every five pathways as explanatory variables according to the feature importance rankings. From the top five pathways, 96 gene expression levels were selected as explanatory variables. This logistic regression model had an accuracy of 0.931, precision of 0.933, recall of 0.922, and F1 score of 0.930 for the test dataset. The F1 scores of the logistic regression models with the genes in every five pathways as explanatory variables are shown in Fig 5A. The model with the genes in the top five pathways had an F1 score of 0.930, while the model with the genes in the bottom five pathways had an F1 score of 0.700. Additionally, the logistic regression classifier with the 367 genes included in the five pathways with the highest absolute normalized enrichment scores was trained and showed an accuracy of 0.791, precision of 0.696, recall of 1.000, and F1 score of 0.821 on the test dataset. Genes in the enriched pathways showed low discriminative ability. The classification performance of the logistic regression classifier with the top 100 gene expression levels in SHAP as explanatory variables had an accuracy of 0.934, precision of 0.974, recall of 0.886, and F1 score of 0.928 for the test dataset. The F1 score of the logistic regression model based on the rank of Shapley values is shown in Fig 5B. The F1 score was 0.928 for the top 100 genes, but decreased to 0.649 for the bottom genes. The classification performance of the logistic regression classifier declined according to the rank of the feature importance for variables.

## Discussion

We investigated whether the Shapley values of pathways in our GCN model represented the feature importance for predicting DLBCL subtypes from gene expression profiling. The graph networks in our model were based on KEGG pathways; therefore, the outputs of graph convolution corresponded to the features in which gene expression patterns contributed to predicting the phenotypes. Highly important pathways according to SHAP included those with high normalized enrichment scores in GSEA. Their Shapley values were considered to indicate the importance of each pathway in phenotype prediction.

The GCN model performed well on the test dataset. The MLP model, which fitted best on the training dataset, showed worse classification performance on the test dataset compared to that of the GCN. The difference in the classification performance between the training and test datasets may be related to differences between the datasets or batch effects [33], as well as from over-fitting to the training dataset. The GCN model, for which graph convolutions were performed to extract the feature from related genes, was expected to make more robust predictions compared to those of the MLP.

There were common pathways selected by both SHAP and GSEA. The glycosaminoglycan synthesis pathway, which was ranked high in both analyses, was associated with the subtypes and aggressiveness of DLBCL [34–36]. In addition, the TGF-β signaling pathway, which is related to apoptosis, was ranked high in both analyses. Genes in this pathway are downregulated in patients with DLBCL, and loss of this signaling induces germinal center B-cell proliferation [22, 37]. In contrast, 13 pathways were selected only by SHAP. The B cell receptor signaling pathway was ranked high only in SHAP. B-cell receptor signaling activates NF-κB, which is involved in B-cell differentiation, proliferation, and survival via Bruton’s tyrosine kinase-dependent phosphorylation. It may be a therapeutic target of B-cell lymphocytic malignancy [38]. The NF-κB pathway is highly expressed in ABC [21, 38, 39]. Therefore, the features extracted from this pathway in the GCN model could detect correlations with phenotypes that were not obvious in GSEA. Biosynthesis of unsaturated fatty acids has been linked to genes whose expression is regulated through methylation of SHMT2, which is highly expressed in GCB [40]. Furthermore, dysregulation of fatty acids has been observed in the subtypes of lymphoid malignancy. Fatty acid synthase is overexpressed in multiple myeloma, a malignancy of the mature B-cell lineage, although changes in the DLBCL subtypes are unknown [41]. Some pathways may highly contribute to the prediction because they contain genes with high Shapley values. Limonene and pinene degradation and riboflavin metabolism included EHHADH and ENPP3, which showed the high Shapley values, respectively, although their biological relevance remains unclear.

GSEA reveals whether gene expression levels are biased from the correlation between the expression levels in gene sets in the annotated pathways and phenotypes. The ratios of expression levels by phenotypes are generally used to calculate enrichment scores. The correlation between GSEA and SHAP was investigated previously. Yap et al. applied SHAP to a convolutional neural network model designed to classify 47 tissue types from transcriptome data [42]. The frequency of genes selected by SHAP in genetic pathways corresponded to the GSEA results. In this study, features corresponding to the phenotypes were obtained by graph convolution, where gene sets and their relationships were used as graphs in the GCN model. Next, the two subtypes were classified based on these features. The trainable weights in the graph convolution layers were fitted to the classification task by training. The features obtained through graph convolution included the complex expression correlation of the pathways and subtypes. Therefore, it may be possible to detect pathways associated with phenotypes that were not detected by GSEA. Other graph convolution techniques, such as relational graph convolution networks [43], focus on the association type of nodes. These new techniques may help create networks by differentially processing various genetic interactions, such as activation and inhibition, although these techniques were not used in this study. Additionally, information obtained using these techniques may be used as directed graphs of gene pathways based on more detailed gene interactions.

The gene expression levels for which pathways included high mean absolute Shapley values were used as explanatory variables for the logistic regression classifier to validate the association between feature importance and predictive performance. This model showed better classification performance than the logistic regression model that used gene expression levels for which pathways with low mean absolute Shapley values were included. This suggests that SHAP applied to the intermediate layer of GCNs was useful for presenting pathways that are strongly associated with phenotypes. Similarly, the logistic regression classifier that used the gene expression levels with high absolute Shapley values showed better classification performance compared to the model that used gene expression levels with low absolute Shapley values. Several discriminative genes have been reported in previous studies [26, 44]. Genes specifically expressed according to the stage of B cell maturation were used in Bayesian classifiers to classify the DLBCL subtypes as the golden standard in the datasets [19, 25–27]. Additionally, immunohistochemistry of CD10, MUM1, BCL6, FOXP1, and GCET1 is useful for distinguishing between GCB and non-GCB [27, 44, 45]. Although few of these genes were contained in the KEGG pathways, genes selected by SHAP also showed high classification performance for DLBCL subtypes.

This study had some limitations. First, the classification performance of the GCN was inferior to that of the logistic regression model. Although the KEGG pathway database was comprehensively manually annotated [29], the graphs of genetic interactions were not specific to the task. The classification performance can be improved by updating the annotated genetic interaction database. A recent study that reported good classification performance for the breast cancer subtype by GCN used a protein-protein interaction network determined using STRING to construct a graph network [16, 46]. However, the limitations of graph convolution in extracting features for accurate prediction have also been pointed out [47]. To apply SHAP to graph networks representing pathways, other pathway databases such as REACTOME [48] and Gene Ontology [49] may be useful. However, these pathways require a larger graph size than KEGG pathways as well as many parameters to implement the model. Because of the small sample size, these pathway databases were not selected in this study. In GSEA, the precision of the prediction depends on the quality of gene set databases [50]. Furthermore, although SHAP provided the feature importances of pathways and gene expression levels and enabled the comparison of these values with other analysis methods, there is no valid method for obtaining confidence intervals and FDR for pathways as in GSEA. Further research is needed to establish the use of SHAP and deep learning models in genetic analyses. Because unclassifiable samples were the remaining clusters that were not classified as GCB or ABC, they were excluded from analysis. Other classification should be used, or further biological studies should be performed to determine the unknown characteristics to address these clusters.

In conclusion, we implemented the GCN with the graphs representing genetic pathways to evaluate the feature importance of pathways by SHAP that contributed to the classification of DLBCL subtypes. The model revealed high-contribution pathways in common with GSEA, and the top-ranked pathways showed high classification performance when they were used as explanatory variables in logistic regression.

## Supporting information

S1 Fig Distribution of the node numbers and edge density. A. Distribution of the node numbers in the 186 KEGG pathways. The horizontal axis shows the number of genes in the pathway and the vertical axis shows the number of pathways. B. Distribution of the node numbers in the top 20 pathways in SHAP. C. Distribution of the node numbers in the top 20 pathways in GSEA. D. Edge density of the 186 KEGG pathways. Edge density is the percentage of edge numbers out of all possible edge combinations in a graph. The histograms show the number of graphs according to the edge density. E. Edge density of the top 20 pathways in SHAP. F. Edge density of the top 20 pathways in GSEA.
(EPS)
